# Osteoid Osteoma in Children Younger than 3 Years of Age

**DOI:** 10.1155/2019/8201639

**Published:** 2019-09-09

**Authors:** Nikolaos Laliotis, Chrysanthos Chrysanthou, Panagiotis Konstantinidis, Lizeta Papadopoulou

**Affiliations:** Interbalkan Medical Center, Thessaloniki, Greece

## Abstract

We present a case series of four children, younger than 3 years old, with osteoid osteoma of the lower limb. Pain and limping were the main symptoms. With careful clinical examination, we could localize the affected area. Radiological evaluation revealed cortical thickening in 3 children. On MRI examination, we found extensive edema, with normal bony cortices. The central nidus was found in 3 children. CT scan was the most accurate examination which revealed the central nidus with surrounding sclerosis. Bone scans had positive uptake in the affected area. Our patients were treated with an intralesional excision biopsy, with simultaneous radiofrequency ablation in those affected in the femur. Pathological specimens confirmed the diagnosis of osteoid osteoma. There was uneventful recovery of our patients. This case series contributes to the limited description of osteoid osteoma diagnosed and treated in very young children.

## 1. Introduction

Osteoid osteoma (OO) mainly affects patients in the second and third decades of life. It is characterized by a central nidus, which consists of osteoid tissue, surrounded by a reactive sclerotic bone with elements of inflammation. Pain usually occurs at night and can be relieved by nonsteroidal anti-inflammatory medication. When the lesion is located in the leg, the child presents with limping. Osteoid osteoma has been found to affect all bones, but it is more common in the long bones, specifically in the femur, tibia, and humerus. Previous reports which identify OO in children younger than 3 are only sporadic [1, 2]. In a large series for OO in the literature, the age at the time of diagnosis ranged from 3 to 20 years old [3–10].

A precise diagnosis is required when a child presents with limping and pain, particularly during the night; radiological finding of periosteal thickening; and extensive bone edema in MRI examination. Differential diagnosis usually includes infection, stress fractures, histiocytosis, and most importantly malignant diseases. It is an exception to include osteoid osteoma in the differential diagnosis for this age group [8–10].

During 2007-2018, we treated four patients younger than 3 years with OO. In this document, we report on the clinical and radiological investigation and treatment we provided, in order to draw attention to the presence of OO in this age group.

## 2. Patient Method

We present four children with ages of 18 months to three years old that were diagnosed with osteoid osteoma.

The main symptom was pain that was present both day and night, severe enough to awaken two children in their sleep.

All were limping during the usual activities of their age; however, no significant trauma was reported by their parents. On clinical examination, all children were in good health. Even at their young age, the children were able to localize the area of pain with careful examination. On palpation, one child had painful swelling in the lateral left malleolus. They refused to run and jump. Joint movements were in normal range. In two of them, small atrophy in the muscles of the femur was noticed.

The pain occurred for 3-8 months before the definite diagnosis. Children were relieved of their pain using pediatric anti-inflammatory medication.

The lesion was located in the femur in two children, affecting the lesser trochanter in one child and the diaphysis in the other child. For the other two, one had the lesion in the distal metaphysis of the tibia and the other at the lateral malleolus. None of the lesions were located in the epiphysis.

In all children, a detailed blood test examination was performed. All findings were within normal limits, including ESR, CRP, and alkaline phosphatase.

Three children demonstrated cortical thickening on plain X-ray examination. This was found in the distal tibia, lateral malleolus, and diaphysis of the femur. The X-ray examination for the child affected in the lesser trochanter was normal (AP and frog lateral).

**Figure 1:**
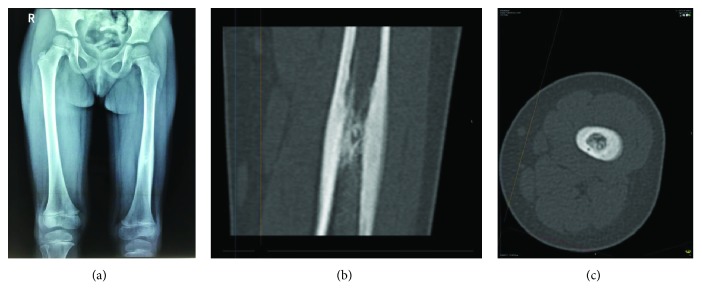
(a–c) Osteoid osteoma of the diaphysis of the left femur. X-ray with cortical thickening and CT with the nidus, surrounded by thickened cortices.

**Figure 2:**
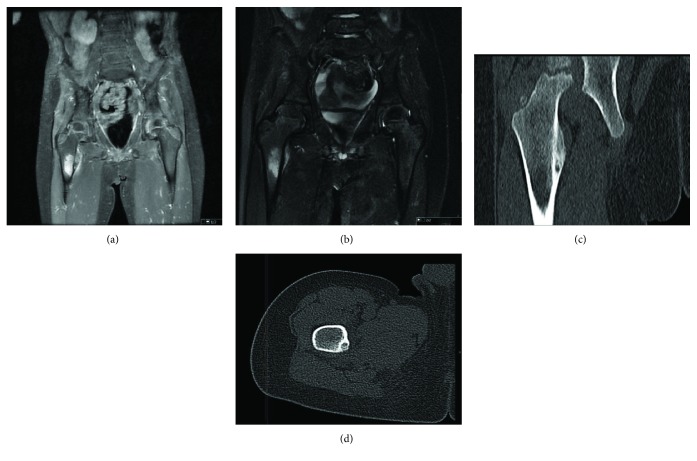
(a, b) MRI with diffuse edema both on T1 with gadolinium uptake and STIR, on the lesion of the lesser trochanter. (c, d) CT scan with the nidus in the lesser trochanter.

The children were further examined with MRI. In all our patients, we found diffuse edema, both intramedullary and in the periosteal area. There was smooth cortical thickening, without scalloping. The central nidus was localized in 3 children. An exception occurred in the child with the lesion in the lesser trochanter, where only diffuse edema was diagnosed (Figures 1(a)–1(c) and 2(a)–2(d)).

A CT scan was performed at the same time, completing the examination process, where only the area of interest was scanned. In all patients, the central nidus surrounded with sclerotic bone was found.

We performed bone scan with Tc^99^ in all children. In all the patients, we found positive uptake in the affected area only.

## 3. Treatment Results

**Figure 3:**
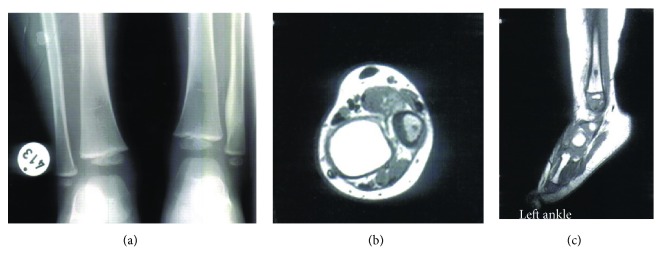
(a) X-ray with thickening of the distal part of the lateral malleolus. (b, c) MRI with the nidus of the OO on the longitudinal and transverse planes.

The children affected in the tibia and fibula were surgically treated with a minimal open procedure, using CT guidance for accurate location of the lesion. We removed a cylinder of bone using a core biopsy needle. The subcutaneous location of the lesion facilitated the OO removal, avoiding thermal burn for a superficial use of RF coagulation (Figures 3(a)–3(c)).

For the other 2 children affected in the femur, after removal of the cylinder of bone, we proceeded with RF ablation, in case of unsuccessful removal of the nidus.

All specimens were sent for pathology. In all except one of our cases, a typical central nidus from osteoid tissue was found, surrounded from reactive bone. The amount of bone removed from the child with the lesion in the lesser trochanter was referred to as insufficient to confirm with confidence the diagnosis of a nidus.

There was an uneventful recovery. All children were symptom free in a short time and returned to their normal activities. We reviewed all patients at 2, 6, and 12 months post treatment. They had a new MRI one year after the procedure. Bone edema had disappeared in 3 children. It remained in a much smaller area, in the child with the lesion of the lesser trochanter.

## 4. Discussion

Osteoid osteoma causes pain and limping when localized in the lower extremity. When dealing with preschool children where pain and limping are prolonged, a thorough investigation is required. It is difficult for a toddler to accurately localize the area of pain. In our series of patients, careful clinical examination showed the exact area of pathology in the lower extremity. Localized tenderness was the most helpful sign. The location of the lesion in the tibia and fibula results in a more accurate localization, but even with lesion in the hip or in the femur, it was possible to find the area of pain.

Osteoid osteoma is most commonly located in the long bones but has been found in all parts of the skeleton in children. The lesion is rare in children younger than 3 years old.

Lindner et al. [3] refer to a series of 58 patients with starting age of 3 years. Kneisl and Simon [4] report 24 patients, the age again ranging from 3 years.

In the series of Sluga et al. [5], they described 125 patients, ages between 3 and 49 yrs old. In the series of Rosenthal et al. [6] in 125 patients, they report one girl 3 yrs old with OO of the femur. Recently, Erol et al. [7] report that 47 children with OO had a range of ages 4-19 years old. Song et al. [8] report on 43 children with range 4.2 to 15.9 for the boys and 6.2 to 13.5 years for the girls. In a most recent series from Napoli et al. [9], with 53 patients with OO, the age of patients ranged from 4 to 45 years old. Hage et al. [10] referred to 92 patients, with starting age for the pediatric population from 4 to 17 years old.

Sporadic cases for OO in children younger than 3 yrs have been reported.

Bhat et al. [2] reported treatment of OO in a 27-month-old child and Haberman and Stern [1] in an eight-month-old boy. Osteomyelitis was strongly suggestive from the CT scan, because there was a small irregular sclerotic area within the lesion that was thought to be a sequestrum rather than a nidus. A biopsy was used to confirm the diagnosis of OO.

Virayavanich et al. reported [11] an OO of the femur in a 7-month-old infant upon MRI examination. Without precise diagnosis, the child underwent CT core needle biopsy that confirmed OO as the final diagnosis. The infant after that had a RF procedure for the treatment. The authors comment on the difficulties to identify the nidus on MRI.

Ekström et al. [12] reported OO in a 1-year-old boy, in the distal part of the femur, confused for possible osteomyelitis because of simultaneous fever. The MRI showed an ill-defined extra- and intraosseal edema without tumor component limiting the differential diagnosis to osteomyelitis, histiocytosis, or OO. Therefore, an open biopsy was performed, and histological examination showed OO. There was partial removal of the nidus, and the procedure was then completed with RF ablation.

Falappa et al. [13] reported on 11 patients with OO, younger than 6 years. They presented a 16-month-old boy and a 3-year-old girl with OO in the tibia. Their initial images suggested osteomyelitis; however, upon reviewing the images, OO was finally diagnosed and confirmed with bone scintigraphy. They were treated with a cooled probe tip.

MRI examination is usually performed after the initial X-ray examination, despite that it requires anesthesia, in order to investigate cortical thickening. In addition to localization of the lesion, our patients showed extensive bone edema both intramedullary and in the surrounding tissues. In cases of infection or malignancy, cortical destruction and periosteal elevation will be present. MRI does not always reveal the central nidus of the OO, as with our case in the lesser trochanter.

MRI shows low intensity on T1 and an increase on T2 weighted images and high contrast enhancement after gadolinium injection. In up to 35% of cases, the nidus cannot be detected, as it is hidden from the surrounding edema [14–16].

Small lesions may be difficult to identify on MRI because the nidus signal is often similar to that of the surrounding cortex.

The use of MRI is helpful but not diagnostic, even for difficult localization of OO, for example, the elbow and hip regions [17–20].

A CT scan can accurately demonstrate the presence of a central nidus, which is the method of choice in almost all series in the literature. There is usually a well-defined round or oval lesion, surrounded by osteosclerosis. In our cases, using CT, we were able to detect the central nidus in all our patients. We recommend starting with a CT scan, when OO is a possible diagnosis, than to begin with MRI. Diffuse edema that is found in MRI examination may be a misleading sign for the correct diagnosis. Dynamic contrast-enhanced CT is helpful to differentiate cases of chronic osteomyelitis and Brodie abscess [14, 15, 21].

All the bone scans in our patients were positive. Uptake is characteristic in cases of OO and can further add for accurate localization but is also positive in cases of fractures or malignancy.

The presence of periosteal reaction when combined with pain, mainly night pain, is important to be differentiated from fracture, infection, chronic osteomyelitis, and tumors. Stress fractures in children occasionally may require a combination of MRI and CT, when they create a diagnostic dilemma. As in cases of OO, there is marked edema in the bone and the surrounding tissues in MRI, but the fracture line can be visualized either in MRI or CT, confirming the diagnosis of fracture [22].

When a child younger than 3 yrs presents with pain and limping, OO is not usually included in the differential diagnosis. Despite this, OO was the most apparent diagnosis in our patients. When completing all examinations, their age made us uncertain, until the diagnosis was confirmed from the pathological specimen [23, 24].

During the last decade, the method of choice of treatment for OO is with percutaneous radiofrequency coagulation [25–29]. In order to have a pathological specimen, removal of the nidus with core bone biopsy and CT guidance is required. We preferred this method for the children with a subcutaneous lesion in the anterior part of the tibia and at the lateral malleolus. Thermal lesions have been described for the subcutaneous lesions, when using RF coagulation. Regarding the lesions of the femur, we completed our procedures with RF ablation, after the removal of the bone. Minimal invasive excision of OO was reported with 3-6 cm incision for the femur and gradual removal of sclerotic bone until visualization of the small nidus that appears as a red spot [7]. We preferred CT-guided biopsy as it has even less bone exposure and is safe for the subcutaneous lesions.

The final treatment is complete removal of the nidus as recurrence is usually due to insufficient removal of the nidus. Occasionally, it is very difficult to localize the small area of pathology.

## 5. Conclusion

Osteoid osteoma is a pathological entity that even occurs in toddlers. The diagnostic approach used to detect pain and limping in preschool children is described. MRI, CT scan, and bone scan were the appropriate examinations for the correct diagnosis. Surgical treatment with removal of the nidus and RF ablation were used for this age group.
